# An antigen processing and presentation signature for prognostic evaluation and immunotherapy selection in advanced gastric cancer

**DOI:** 10.3389/fimmu.2022.992060

**Published:** 2022-10-14

**Authors:** Ke-wei Wang, Mei-dan Wang, Zi-xi Li, Ben-shun Hu, Jun-jie Wu, Zheng-dong Yuan, Xiao-long Wu, Qin-fang Yuan, Feng-lai Yuan

**Affiliations:** ^1^ Institute of Integrated Traditional Chinese and Western Medicine, Affiliated Hospital of Jiangnan University, Wuxi, China; ^2^ Department of Hepatobiliary Surgery, Affiliated Hospital of Jiangnan University, Wuxi, China; ^3^ Department of hospital infection, Affiliated Hospital of Jiangnan University, Wuxi, China

**Keywords:** antigen processing and presentation, immune checkpoint inhibitor, immunotherapy, tumor microenvironment, survival

## Abstract

**Objective:**

The aim of the study was to propose a signature based on genes associated with antigen processing and presentation (APscore) to predict prognosis and response to immune checkpoint inhibitors (ICIs) in advanced gastric cancer (aGC).

**Background:**

How antigen presentation-related genes affected the immunotherapy response and whether they could predict the clinical outcomes of the immune checkpoint inhibitor (ICI) in aGC remain largely unknown.

**Methods:**

In this study, an aGC cohort (Kim cohort, RNAseq, N=45) treated by ICIs, and 467 aGC patients from seven cohorts were conducted to investigate the value of the APscore predicting the prognosis and response to ICIs. Subsequently, the associations of the APscore with the tumor microenvironment (TME), molecular characteristics, clinical features, and somatic mutation variants in aGC were assessed. The area under the receiver operating characteristic curve (AUROC) of the APscore was analyzed to estimate response to ICIs. Cox regression or Log-rank test was used to estimate the prognosis of aGC patients.

**Results:**

The APscore constructed by principal component analysis algorithms was an effective predictive biomarker of the response to ICIs in the Kim cohort and 467 aGC patients (Kim: AUC =0.85, 95% CI: 0.69–1.00; 467 aGC: AUC =0.69, 95% CI: 0.63–0.74). The APscore also was a prognostic biomarker in 467 aGC patients (HR=1.73, 95% CI: 1.21−2.46). Inhibitory immunity, decreased TMB and low stromal scores were observed in the high APscore group, while activation of immunity, increased TMB, and high stromal scores were observed in the low APscore group. Next, we evaluated the value of several central genes in predicting the prognosis and response to ICIs in aGC patients, and verified them using immunogenic, transcriptomic, genomic, and multi-omics methods. Lastly, a predictive model built successfully discriminated patients with vs. without immunotherapy response and predicted the survival of aGC patients.

**Conclusions:**

The APscore was a new biomarker for identifying high-risk aGC patients and patients with responses to ICIs. Exploration of the APscore and hub genes in multi-omics GC data may guide treatment decisions.

## Introduction

The morbidity and mortality of gastric cancer (GC) rank fifth and fourth among all malignant tumors (1). In recent years, immune checkpoint inhibitors (ICIs), such as anti-cytotoxic T-lymphocyte-associated protein 4 (CTLA-4) inhibitors and anti-programmed death 1 (PD-1)/programmed death-ligand 1 (PD-L1) inhibitors, in combination with chemotherapy and immunotherapy have made significant progress in multiple types of cancers (2). Several ICIs have been approved for the clinical treatment of advanced GC (aGC) patients (3, 4). But the overall response to ICIs is only 20% to 40% for GC patients (2, 5). So it is urgent to accurately identify effective biomarkers to select GC patients with responses to ICIs.

The tumor mutation burden (TMB), neoantigen load and clonality, copy number alterations (CNA), microsatellite instability (MSI) status, tumor microenvironment (TME), especially T cell inflammation, PD-L1 expression, and mutations in specific genes are now deemed to be predictive markers for immunotherapy in aGC patients. These indicators do, however, have limitations that have hindered their clinical application (6–9). Tumor immunogenicity mainly consists of tumor antigenicity and antigen presentation, playing a crucial part in response to ICIs in most types of cancer (10, 11). But accuracy of identifying aGC patients with response to ICIs according to TMB is low (12–14). Tumor antigens from mutated proteins of tumor cells enable tumor cells to be recognized and killed by CD8^+^ T cells through antigen-presentation mechanisms (11, 15). Therefore, antigen presentation significantly affects the effect of ICIs treatment. Nevertheless, we do not find relevant studies on how antigen presentation-related genes affect the immunotherapy response and whether they can predict the clinical effect of ICIs therapy in aGC patients.

Thus, we proposed a signature based on antigen processing and presentation genes to predict prognosis and response to ICI in aGC patients. To the best of our knowledge, this study was the first to explore the role of antigen processing and presentation in aGC and select hub genes to understand progressive tumor mechanisms and offer promising and personalized strategies to diagnose and treat GC.

## Methods

### Data source

The study used GC-related data from two public platforms, the Cancer Genome Atlas (TCGA) Genomic Data Commons Data Portal (https://portal.gdc.cancer.gov/) and the Gene Expression Omnibus database (GEO) (https://www.ncbi.nlm.nih.gov/geo/). TCGA provided mRNA expression, copy number mutation, and clinicopathologic profiles including age, gender, Lauren type, tumor grade, tumor stage, and survival. GEO provided six GC-related cohorts including GSE84437 (16), GSE57303 (17), GSE34942 (18), GSE29272 (19), GSE15459 (20) and ACRG/GSE62254 (21). The aGC was defined as the presence of metastatic disease or tumor stages higher than IV. Patients without any survival data as well as those with less stage IV or primary tumors were excluded. The final merged aGC cohort (FMAC) consisted of 467 patients, encompassing 103 from the TCGA-STAD dataset and 364 from six GEO datasets. All gene expression or transcriptome data were transformed with log2 (x +1) and then were batch rectified by SVA Package of R (22).

Next, this study included two cohorts of cancer patients treated with immunotherapy. 45 aGC patients treated with ICIs in Korea (5) (The Kim cohort, PRJEB25780, https://www.ebi.ac.uk/ena/data/view/PRJEB25780) were included in the first cohort. The cohort collected some data about RNAseq data, immunotherapy regimens, response to ICIs, TCGA subtype, microsatellite instability (MSI), Epstein‐Barr virus (EBV), Mesenchymal subtype, single nucleotide variants (SNVs) and immune signature (5). The second cohort, the IMvigor210 cohort (23) (http://research-pub.gene.com/IMvigor210CoreBiologies) of bladder cancer (BC), provided gene expression data, survival, immunotherapy regimens and response. Partial response (PR) and complete response (CR) to ICIs were considered to be immunotherapy responses (responses to ICIs). Moreover, patients without immunotherapy responses included those with progressive disease (PD) and stable disease (SD).

### Weighted gene co-expression network analysis

To identify antigen processing and presentation genes related to immunotherapy response, WGCNA package of R was used to calculate Pearson correlation coefficient among genes, and select an appropriate soft threshold β according to the scale-free topological fitting index of R^2^ >0.9 and average connectivity (24). So, the constructed network could be in line with the standard of scale-free network.

Next, the gene network was constructed by one-step method, and the adjacency matrix of expression data was transformed into a topological overlap matrix. The hierarchical clustering method was used to plot the hierarchical cluster tree to cluster genes into different color modules. The feature values of gene modules were calculated, and then Pearson correlation coefficient and correlation between the feature vector and clinical information were calculated. The gene set modules with the highest correlation with immunotherapy response, antigen processing and presentation were selected for further analysis. We first calculated the eigenvalues of gene modules, and then calculated the correlation coefficients between the feature vectors of the modules and clinical features (immunotherapy and antigen processing and presentation). The gene set modules with significant correlation with immunotherapy response and antigen processing and presentation were available for further analysis.

### Analysis of biological function and pathways of genes

The Gene Ontology (GO; molecular function, cellular component, and biological process) database and informatics resource (http://www.geneontology.org) were used to annotate gene function enrichment. Kyoto Encyclopedia of Genes and Genomes (KEGG) database (http://www.genome.ad.jpl/kegg/) with analysis of cells or organisms senior functional behavior was used to annotate various pathways enrichment of genes. Additionally, the identification of key pathways enriched by hub genes was conducted by the GSCALite(http://bioinfo.life.hust.edu.cn/web/GSCALite/) and the Metascape web server(http://metascape.org/) (25, 26).

### Construction of APscore

The WGCNA selected genes associated with immunotherapy response and antigen processing and presentation for further analysis. Above genes often involved hundreds of genes, of which significant interrelationships were inevitable. To better analyze the expression data of these genes, we synthesized multiple variables with multiple correlations into several representative variables, representing the majority of the original variables’ information but unrelated to each other. Therefore, we performed principal component analysis (PCA) to reduce the dimension of the above genes expression data set (27). Then we obtained principal component 1 and 2, of which the sum was defined as the APscore (28). The specific formula is as follows: APscore= $\sum^{}(PC1_{i}+PC2_{i})$ , where i represents the expression of genes. PCA scores for some important tumor-related pathways including antigen processing machinery, immune checkpoint, CD8 T effector, DNA damage repair, epithelial-mesenchymal transition (EMT) and so on were calculated according to the expression of genes enriched in these pathways (29–31).

### Assessment of immune microenvironment and immunotherapy response

Twenty-two immune cell invasions according to gene expression were assessed by the CIBERSORT algorithm (32). According to gene expression, the absolute abundance of eight immune and two stromal cell populations were evaluated by the microenvironment cell populations-counter (MCP-counter) method (33). The ESTIMATED algorithm assessed each patient’s immune and stromal scores according to gene expression (34). The TIDE algorithm platform was used to evaluate immunotherapy response according to standardized gene expression in FMAC (35).

### Selection of hub genes association with prognosis and immunotherapy response

The ten most crucial hub genes associated with immunotherapy response and antigen processing and presentation were identified using the MCODE and Cytohubba of the Cytoscape software (36, 37). Then we assessed the efficacy of hub genes predicting prognosis in FMAC and immunotherapy response in the Kim cohort. The predictive efficacy was also validated in another aGC cohort (GSE26253) treated with chemoradiotherapy (38).

### Construction of predictive model for prognosis and immunotherapy response

First, we selected target differently expressed genes (DEGs) that were associated with immunotherapy response and enriched in the antigen processing and presentation in the Kim cohort. Second, those target DEGs were intersected with genes from FMAC, and then shared genes were obtained and used to construct the predictive model for prognosis by Cox regression in FMAC and for immunotherapy response by logistics regression in the Kim cohort. Last, the risk score of the predictive model was calculated based on the coefficient of Cox regression or logistics regression and gene expression. The specific formula is as follows:


$$risk\;score=\sum^{}\left(expression\;of\;gene_{i}\times coefficient_{i}\right)$$

### Construction of nomogram for prognosis

We used Cox regression to select some independent variables including several signaling pathways, the risk score of the predictive model for prognosis and immunotherapy response, and the ratio of M1 Macrophages to M2 macrophages, which had significant associations with the prognosis of GC patients. According to the above independent variables, we built a nomogram to predict the survival of aGC patients. Additionally, each patient’s risk score and 3, 5 and 8-year survival were calculated.

The Cox regression was conducted by rms or survival package of R; the nomogram was plotted by regplot package of R (39). The timeROC package of R was used to plot receiver operating characteristics (ROC) curves for 3, 5 and 8-year survival, respectively (40). The calibration and decision curves were plotted by the rms package and rmda of R, respectively.

### Specimen collection

Twenty-five patients with GC who underwent surgical resection in the Affiliated Hospital of Jiangnan University were collected. None of the patients received any chemotherapy, radiotherapy or biotherapy before surgery. This study was approved by the ethics committee of affiliated hospital of Jiangnan University and followed the guidelines of the Declaration of Helsinki. Informed consent was obtained from all participants. All specimens were diagnosed as GC by two independent pathological diagnosis doctors. All samples were immersed in formalin solution and fixed for paraffin embedding (FFPE). 4 μm sections were prepared for immunohistochemical staining.

### Hematoxylin-eosin staining

The FFPE section was dewaxed with xylene. Xylene was then deoxidized with gradient alcohol. The tissue was stained for 3min by Hematoxylin. The nuclear staining was observed under the microscope. The tissue was further stained for 90s by the Eosin solution, and the staining was examined under the microscope.

### Immuno-histochemistry

Quantitative analysis methods performed IHC and multi-color immunofluorescence. First, FFPE was dewaxed and hydrated, and the EDTA method repaired tissue antigen. H_2_O_2_ was used to inactivate endogenous peroxidase in tissues. Second, the tissue was covered entirely with primary antibody solution and placed in a wet box at 4°C for 12 hours. Then, the tissue retemperature at room temperature for 30min. The second antibody (horseradish peroxidase-labeled) was added and incubated for 60 min at room temperature for Tetramethylbenzidine color rendering. An optical microscope observed the brown-yellow particles as a positive color reaction.

The primary antibody used in this study: CK (ab52625, Abcam, 1: 1000), CD8 (14-0081-82, Invitrogen Antibodies, 1:200), PD-L1(ab205921, Abcam PD-L1(ab205921, Abcam, 1: 1000), STAT1: (ab29045, Abcam, 1:2000), IFIT3 (ab76818, Abcam, 1:000), TAPBM (ab13518:Abcam, 1: 400). Immune classifications of GC were defined by the CD8^+^ T cells infiltrating the extent and number of tissues (41, 42). The immune-inflamed subtype was characterized by CD8^+^ T cells spread throughout the tumor parenchyma and surrounding stroma. The excluded-immune subtype was characterized by CD8^+^ T cells infiltration in the peritumor stroma, not in the parenchyma. The deserted-immune subtype was characterized by the absence of CD8^+^ T cells in tumor parenchyma and stroma.

### Multi-color immunofluorescence

After being dewaxed, the (FFPE section was repaired by EDTA antigen repair buffer (PH 9.0). The tissues were added with PBS (PH7.4) and primary antibody and incubated overnight at 4°C. The tissues were then covered with secondary antibodies and incubated at room temperature for 50min. DAPI dye was dropped and incubated for 10min at room temperature, away from light. The slices were briefly shaken dry and sealed with anti-fluorescence quenching sealing tablets. The fluorescence microscope was used to observe the protein’s location, shape and quantity, and images were collected.

### Statistical analysis

The t-test or Wilcoxon signed-rank test was used to compare two groups of continuous variables. One-way analysis of variance or Kruskal-Wallis H test was used for comparison among multiple (n>2) groups. The chi-square test or nonparametric rank-sum test was used to compare categorical variables. Pearson or Spearman correlation analysis conducted correlation analysis of two continuous variables. Kaplan-Meiers curve was used to show the survival of the sample over time, and the log-rank test was used to test whether there were significant differences in the survival of multiple groups. The Cox regression was used to select factors affecting survival. Logistic regression was used to screen factors affecting immunotherapy response. All statistical analysis was conducted in R software (version 4.0.3). All analyses were two-sided and tested at the nominal 0.05 significance level.

## Results

### Selection of key modules associated with prognosis and response to ICIs

**Figure 1:**
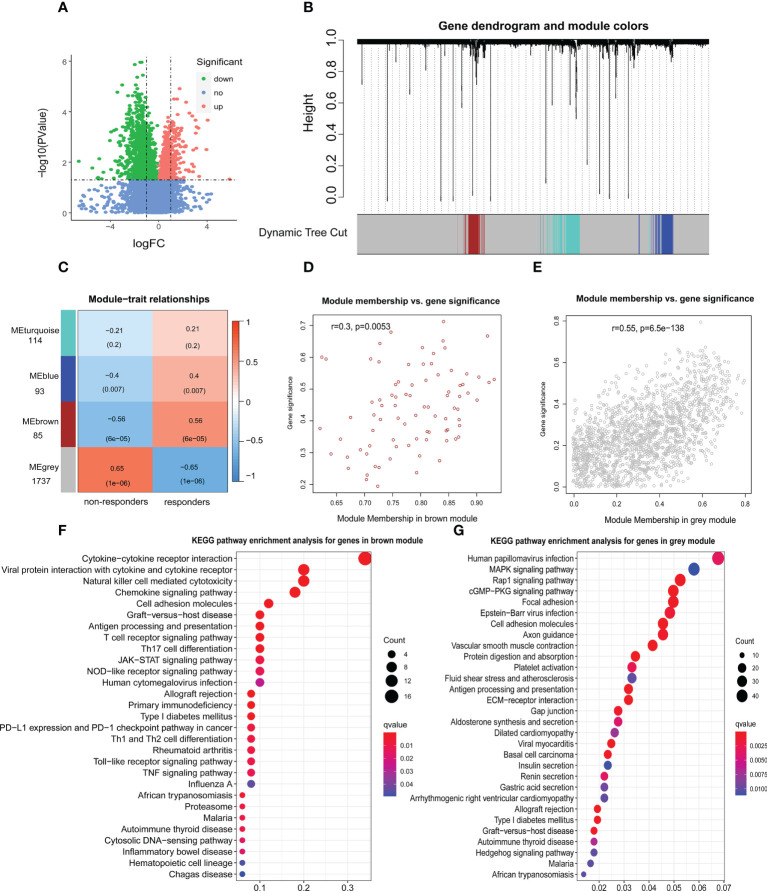
Identification of genes associated with immunotherapy response. **(A)** The volcano figure of differentially expressed genes (DEGs) associated with immunotherapy response in the Kim cohort. logFC: log-fold changes. **(B)** Weighted gene coexpression network analysis (WGCNA) of 2759 DEGs with a soft threshold β = 26. **(C)** Heat maps showing the gene modules associated with immunotherapy response. The relationship between the gene signature and brown module genes **(D)** or grey module genes **(E)**. Kyoto Encyclopedia of Genes and Genomes (KEGG) pathway enrichment analysis for genes in brown module **(F)** or grey module **(G)**.

We identified 2759 DEGs in patients with and without response to ICIs in the Kim cohort (**Figure 1A**; **Table S1**). According to the optimal soft-thresholding power of 26, WGCNA of these DEGs was conducted (**Figures S1A, B**) and showed that these DEGs were divided into four different modules (**Figures 1B, C**). Of which the blue module (r = 0.4, p = 0.007), the brown module (r =0.56, p = 6e–05) and the grey module (r = −0.65, p = 1e–06) were significantly related with immunotherapy response (**Figure 1C**), respectively. Furthermore, the number of brown module members (n=85) significantly correlated with the gene signature (r=0.3, p=0.005, **Figure 1D**). Similar result was found in the grey module (n=1737, r=0.55, p=6.5e–138; **Figure 1E**). However, there were no significant correlations of the gene signature with the blue module and turquoise module (blue module: r= −0.13, p=0.21; turquoise module: r= −0.078, p=0.35; **Figures S1C, D**).

GO analysis showed that brown module genes were enriched in several immune functions, such as immune receptor activity, major histocompatibility complex (MHC) protein binding, T cell activation and cytokine activity (**Figure S1E**; **Table S2**). Those grey module genes were enriched in the extracellular organization and MHC protein complex functions (**Figure S1F**; **Table S3**). Moreover, KEGG analysis showed that brown module genes were enriched in cytokine-cytokine receptor interaction, antigen processing and presentation, T cell receptor signalling pathway, NOD-like receptor signalling pathway, JAK-STAT signalling pathway, TNF signalling pathway and Toll-like receptor signalling pathway, which affected the occurrence, progression and response to ICIs in multiple types of tumors (29–31) (**Figure 1F**; **Table S4**). Those grey module genes were enriched in the cGMP-PKG signaling pathway, antigen processing and presentation, Gastric acid secretion and Rap1 signaling pathway (**Figure 1G**; **Table S5**). Notably, five brown module genes and 23 grey module genes were enriched in antigen processing and presentation (**Tables S4, 5**), implying that antigen presentation status may significantly contribute to immunotherapy.

Next, of 1822 genes (85 brown and 1737 grey genes), 637 genes (turquoise module) were significantly related to antigen processing and presentation by WGCNA (r=0.54, p=1e–04; **Figures S2A, B**). Furthermore, 637 genes significantly correlated with the gene signature (r=0.56, p=7.3e–54; **Figure S2C**).

### The APscore predicts immunotherapy in the Kim cohort

**Figure 2:**
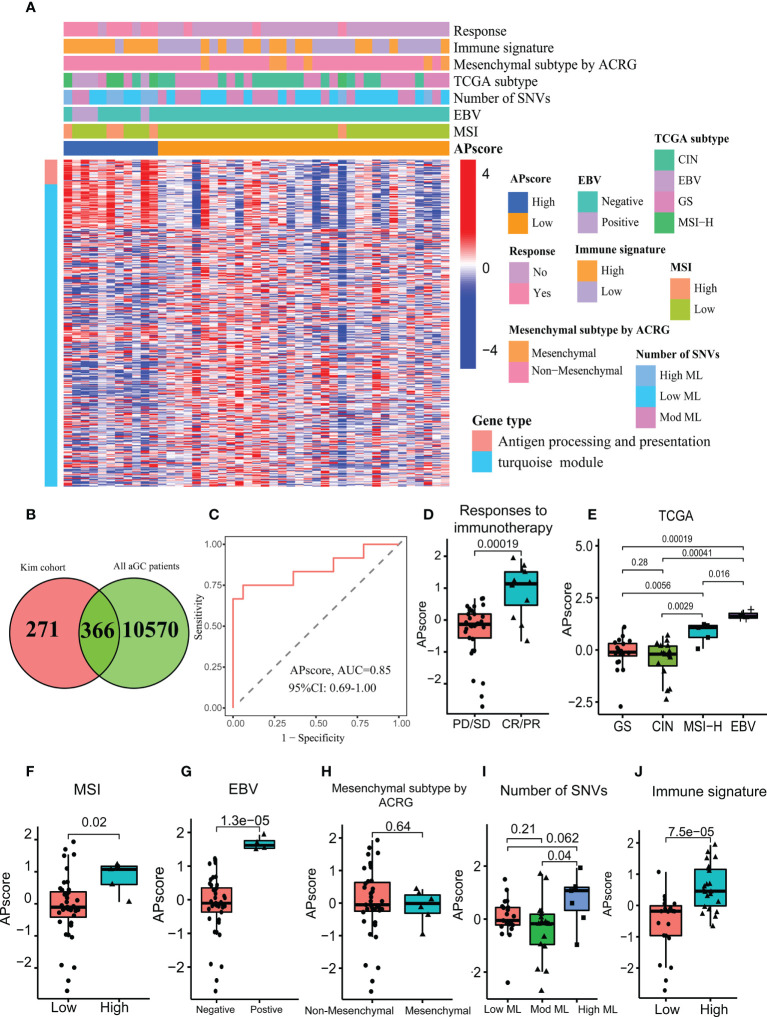
The characteristics of the APscore as an indicator of immunotherapy response in aGC patients. **(A)** The relationship of the APscore with other subtypes of GC. **(B)** Venn showing 366 genes associated with the APscore. **(C)** Receiver operating characteristics (ROC) for the APscore in predicting immunotherapy response. Comparison of the APscore in different groups of responses to immunotherapy **(D)**, TCGA subtypes **(E)**, MSI subtypes **(F)**, EBV subtypes **(G)**, Mesenchymal subtypes **(H)**, Number of SNVs subtypes **(I)** and immune signature subtypes **(J)**.

After intersecting 637 genes above with 10936 genes from FMAC (GSE84437, GSE57303, GSE34942, GSE29272, GSE15459, ACRG/GSE62254 and TCGA-STAD), altogether 366 genes were used to construct a scoring system based on the PCA algorithm, which we called APscore (**Figures 2A, B**). To estimate the relationships of the APscore with target genes, immunotherapy response, immune signature, mesenchymal subtype, TCGA subtype, number of SNVs, EBV subtype and MSI subtype in GC, a combined heat map was plotted (**Figure 2A**).

Based on the cutoff thresholds derived by the Youden index for continuous variables—the APscores of all patients, each patient was regrouped into the high or low-APscore group (43). It was found that most of the 28 genes enriched in antigen processing and presentation were expressed higher in the high-APscore group than in the low-APscore group. The APscore could distinguish between patients with vs. without response to ICIs [area under curve (AUC) =0.85, 95% CI (confidence interval): 0.69–1.00; **Figure 2C**]). Moreover, higher APscore was observed in patients with immunotherapy response (p=0.00019, **Figure 2D**).

Next, the APscore was significantly different among four TCGA subtypes, two MSI subtypes, two EBV subtypes and three Mesenchymal subtypes (**Figures 2E–I**, all p<0.05). High-APscore patients were likely to have positive EBV status, high MSI status and high mutational load, which were known as beneficial factors of immunotherapy, implying that the APscore may be a valuable indicator of immunotherapy. Similarly, higher APscore was observed in the high immune signature subtype (P= 7.5e–05, **Figure 2J**). The immune signature involved 12 genes, CCL2, CCL3, CCL4, CCL5, CCL8, CXCL9, CXCL10, CXCL11, CXCL13, CL18, CCL19 and CCL21, of which expression levels reflected tumor microenvironment in GC (44). So, it was worth further exploring microenvironment features in different APscore groups.

### Immune microenvironment characteristics of different APscore groups

To assess the immunotherapy response, standardized genes expression of all patients in FMAC were entered into TIDE algorithm platform. The APscore of each patient was calculated based on the standardized expression of 366 genes, which were associated with antigen processing and presentation.

**Figure 3:**
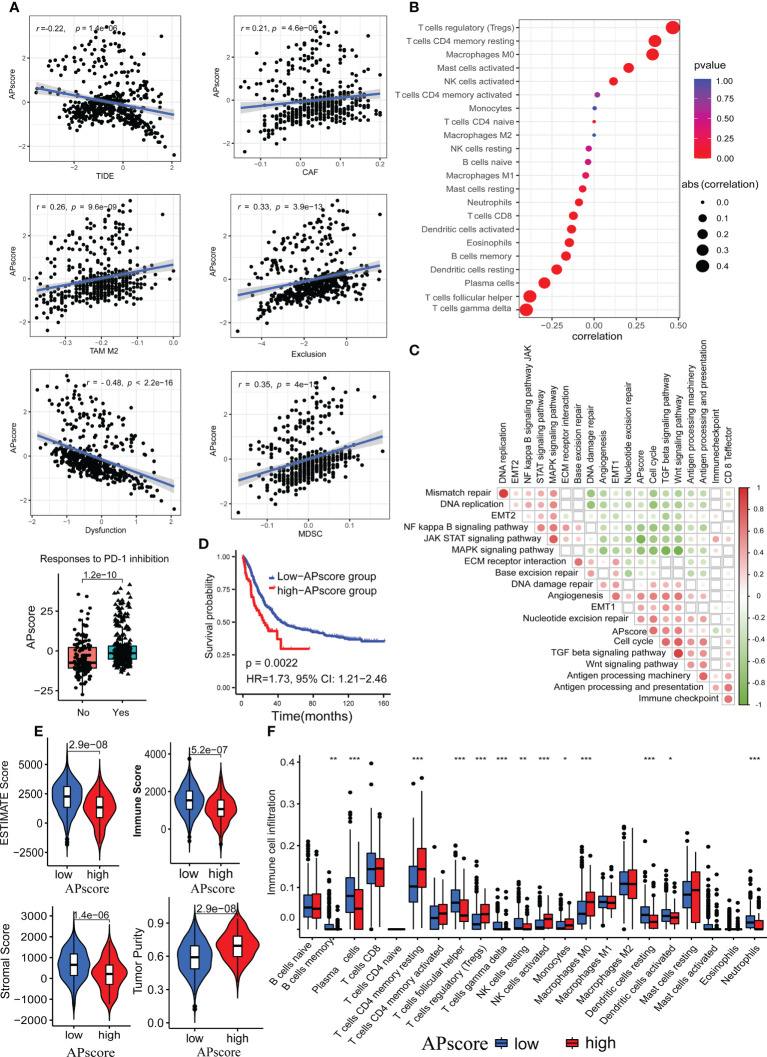
The associations of the APscore with immunotherapy response, immune microenvironment, and survival in aGC patients. **(A)** The relationship of the APscore with indicators of immunotherapy response predicted by the TIDE algorithm. **(B)** The relationship of the APscore with infiltration of the 22 immune cells calculated by the CIBERSORT algorithm. **(C)** The interrelations of the APscore with PCA genes scores of important biological pathways of GC. The white squares represent no statistically significant. **(D)** Kaplan-Meier survival plots in both groups of 467 aGC patients. **(E)** Comparison of the tumor purity, estimate, immune, and stromal scores between the high-APscore and low APscore-groups. **(F)** Comparison of the 22 immune cells proportions between the high-APscore and low APscore-groups. Asterisks denote significant differences (*p< 0.05; **p< 0.01; ***p< 0.001).

The APscore showed significant negative correlations with TIDE, Dysfunction, CD8, Merck18, IFNG and MSI Expr Sig, and positive correlations with CAF, TAM M2, Exclusion and MDSC (all p< 0.05; **Figures 3A**, **S3A**). A high TIDE score represented poor efficacy of immune checkpoint blocking therapy (ICB) and short survival. Moreover, a higher APscore was observed in patients with response to ICIs predicted by the TIDE algorithm (**Figure 3A**, p=1.2e–10), consistent with **Figure 2D**. Expected, the APscore distinguished patients with response to ICIs from patients without response to ICIs in FMAC (AUC=0.69, 95% CI: 0.63–0.74; **Figure S3C**). These results show that the APscore can predict immunotherapy response in aGC patients.

Next, we used the CIBERSORT algorithm to estimate the infiltration of the 22 immune cells. The APscore had significantly positive correlations with regulatory T cells (Tregs), memory resting CD4 T cells, M0 Macrophages, activated mast cells and activated NK cells, and significantly negative correlations with gamma delta T cells, follicular helper T cells, plasma cells, resting dendritic cells, memory B cells, eosinophils, activated dendritic cells, neutrophils and CD8 T cells (all p< 0.05, **Figure 3B**). Of note, the correlations of the APscore with PCA genes scores of crucial biological pathways showed that the APscore had significantly positive correlations with cell cycle, TGF beta signaling pathway, Wnt signaling pathway, antigen processing machinery, antigen processing and presentation, DNA damage repair, angiogenesis, EMT1 and nucleotide excision repair scores, and significantly negative correlations with mismatch repair, DNA replication, EMT2, MAPK signaling pathway, JAK/STAT signaling pathway, NF kappa B signaling pathway, ECM receptor interaction and base excision repair scores (**Figure 3C**, all p< 0.05).

Patients exceeding a certain APscore threshold, defined by the Youden index, were classed as a high-APscore group, the opposite as a low-APscore group. By log-rank test, patients in the high-APscore group had poorer survival than those in the low-APscore group (p=0.0022, HR=1.73, 95% CI: 1.21−2.46, **Figure 3D**). In addition, we used ESTIMATED algorithms to estimate immune and stromal scores for each patient. The estimate, immune, and stromal scores were significantly higher in the low APscore-group than that in the high APscore-group, while the opposite trend was observed for the tumor purity (all p<0.05, **Figure 3E**). Similarly, representative cells for anti-tumor immunity such as NK cells, memory resting CD4 T cells, Tregs, Monocytes and M0 Macrophages were more abundant in the high-APscore group (all p<0.05, **Figure 3F**). These results further verify that the APscore may influence the prognosis and response to ICIs through classifying tumor subtypes according to the tumor microenvironment.

### SNVs of different APscore groups

Previous studies suggest that accumulated somatic mutations induced the immune system to produce anticancer cells, and tumor mutation load affected immunotherapy response and the survival of GC patients (10, 11, 44). To explore the genetic imprints of different APscore groups, we used SNV data of 101 aGC patients from TCGA-STAD to analyze the relationship between the TMB and the APscore. The APscore had a significantly negative correlation with TMB (r=−0.42, p=1.2e–05, **Figure 3SD**). The high-APscore group had lower TMB than the low−APscore group (p=0.001, **Figure 3SE**). Moreover, the high-TMB group had longer survival than the low-TMB group (log-rank test, p = 0.015, **Figure 3SF**). However, we could not find significantly different survival with a p-value of 0.05 among four subgroups formed by the APscore and TMB (p=0.05, **Figure 3SG**). This result may be largely due to limited samples (n=101).

Next, the APscore as a binary variable entered into Cox regression model, and then was an independent prognostic predictor of aGC patients after adjusting other clinicopathologic characteristics, including Lauren subtype, age, gender, cancer grade, CD274 expression, TMB and the ratio of M1 Macrophages to M2 Macrophages (HR=4.42, 95% CI:1.36−14.28, **Figure 3SH**). Moreover, the efficacy of the APscore predicting survival of aGC patients was explored in 14 independently thermal TCGA cohorts including 6673 patients (**Table S6**). The APscore was a significant factor in the prognosis of patients with breast invasive carcinoma (BRCA), head and neck squamous cell carcinoma (HNSC), brain lower grade glioma (LGG) or skin cutaneous melanoma (SKCM), which were generally considered thermal tumors with infiltration by a variety of T cells (**Figure S3I**). Therefore, it needs to explore further the interaction mechanism of the APscore and T-cell infiltration to influence the prognosis.

To assess the driver mutations in different APscore groups, the mutated genes from TCGA-STAD were listed using maftools. In the high-APscore group, the top five mutated genes were TP53, TTN, MUC16, LRP1B, and DNAH5, respectively, whereas the top five were TTN, MUC16, ARID1A, PIK3CA and KMT2D, respectively, in the low-APscore group (**Figure S3J**). Of note, compared with the low-APscore group, the high-APscore group had lower frequencies of mutated genes ARID1A, PCLO, KMT2D, OBSCN and PIK3CA (all p<0.05, **Figure S3J**). A previous study used the TCGA-STAD dataset to estimate the correlation of PIK3CA with sensitivity or resistance (8). Our results provide new insights into the role of the APscore in GC with special emphasis on its effect on individual mutations to offer a reference for the effect of tumor immunotherapy.

### Selection of hub genes associated with prognosis

**Figure 4:**
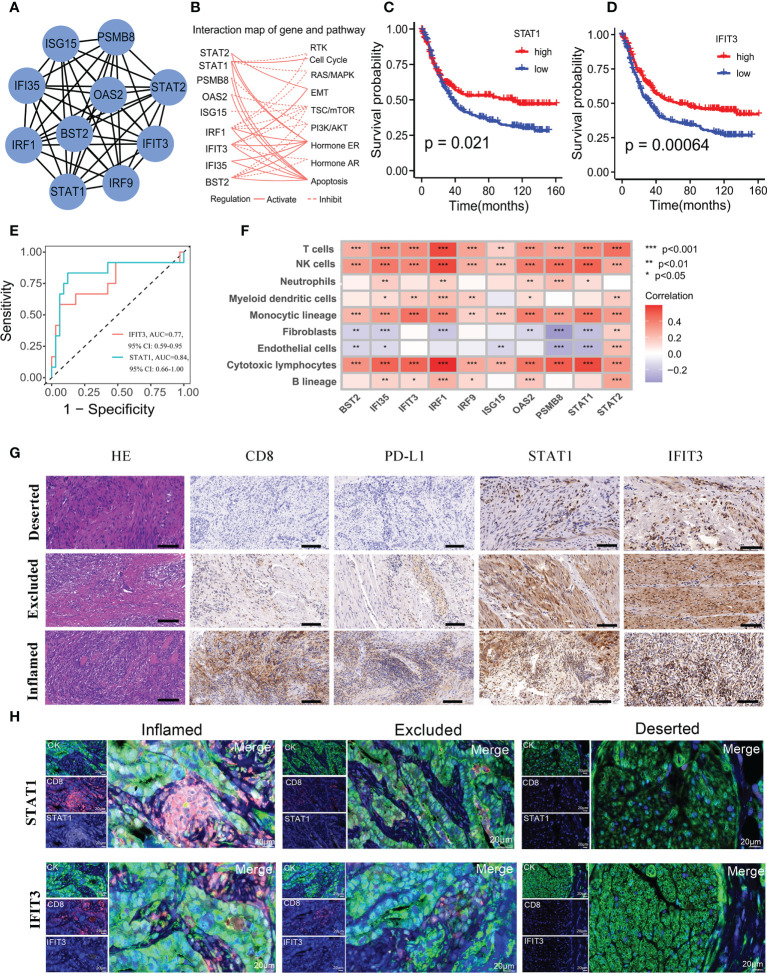
Selection and characteristics of hub genes associated with immunotherapy response and prognosis. **(A)** A network of protein-protein interactions for 10 hub genes. **(B)** The profiles of hub genes affecting canonical signaling pathways. The Kaplan-Meier survival curves of STAT1 **(C)** and IFIT3 **(D)** expression affecting prognosis. **(E)** ROC for the STAT1 and IFIT3 predicting immunotherapy response in the Kim cohort. **(F)** The correlations of hub genes with abundance of seven immune and two stromal cells, calculated by the MCP-counter method. **(G)** Immunohistochemistry detected the expression of CD8, PD-L1, STAT1, and IFIT3 in the immune-inflamed subtype, immune-excluded subtype, and immune-deserted subtype of GC. The scale corresponds to 50μm. **(H)** Multi-color fluorescence staining detected the spatial distribution of STAT1, IFIT3 and CD8 in the immune-inflamed subtype, immune-excluded subtype, and immune-deserted subtype of GC. The scale corresponds to 20μm.

To assess novel targets for GC immunotherapy, we selected the ten most important hub genes (STAT1, IRF1, ISG15, IRF9, BST2, PSMB8, STAT2, IFI35, IFIT3 and OAS2) from above 366 genes being used to generate the APscore. Next, we plotted the interaction map of hub genes and pathways using the Cytoscape software (**Figures 4A, B**). Of note, ten genes were all enriched in interferon-alpha (IFNα)/beta (IFNβ) signaling using the Metascape (**Table S7**). Some studies suggest that IFNα and IFNβ are potential anti-tumor immune cytokines, resulting in improved outcomes for patients with malignancies of heterogeneous histologies (45, 46). In the study, hub genes activated four signaling pathways: cell cycle, EMT, ER Hormone and apoptosis, and inhibited five signaling pathways: RTK, RAS/MAPK, TSC/mTOR, PI3K/AKT and AR Hormone pathway (**Figure 4B**). This result further highlights the potential value of hub genes to predict immunotherapy. It needs to explore the interaction between hub genes and T cells to influence immunotherapy and prognosis in GC.

Next, it was found that ten genes affected the survival of patients in FMAC (**Figures 4C, D**, **4SA**). The expression level of STAT1 and IFIT3 was significantly associated with longer survival (STAT1: log-rank test, p = 0.021; IFIT3: log-rank test, p = 0.00064). In the Kim cohort, patients responding to ICIs had higher expression levels of ten genes than those (all P< 0.05, **Figure S4B**). Intriguingly, STAT1 and IFIT3 distinguished individuals with response to ICIs from individuals without response to ICIs in the Kim cohort (STAT1: AUC =0.84, 95% CI: 0.66−1.00; IFIT3: AUC =0.77, 95% CI: 0.59−0.95; **Figure 4E**). Survival analysis further showed that high expression level of STAT1 and IFIT3 predicted progression-free survival (PFS) benefit for aGC patients in the GSE26253 cohort treated with chemoradiotherapy (all p<0.05; **Figures S4C, D**). In addition, the expression level of STAT1 and IFIT3 was positively related to seven types of immune cells and negatively associated with fibroblasts and endothelial cells calculated by the MCPcounter (p< 0.05, **Figure 4F**; **Table S8**). We observed similar correlations of the expression level of STAT1 and IFIT3 with activated dendritic cells, activated NK cells, CD8 T cells and memory activated CD4 T cells, which were representative cells for anti-tumor immunity estimated by the CIBERSORT. (all p< 0.05; **Figure 4SE**).

To further explore the relationship between the expression of STAT1 and IFIT3 and the immune grade of GC, we selected 25 FFPE samples to quantify the potential relationship between the above two genes and tumor immune microenvironment using HE staining, immunohistochemistry and multi-colour immunofluorescence. The GC was divided into three immune subtypes: immune-inflamed subtype, immune-excluded subtype, immune-deserted subtype according to CD8^+^ T cell infiltration in the tissues (**Figure 4G**). At the same time, the relationship between the two genes and immune cell localization and PD-L1 expression was also shown in **Figure 4G**. It was found that the highest expression of PD-L1, STAT1 and IFIT3 in the immune-inflamed subtype, followed by the immune-excluded subtype and immune-deserted subtype. Moreover, we further experimented with multi-color fluorescence staining to explore the spatial relationship between the two genes and CD8 in different immune subtypes of GC (**Figure 4H**). Expected, STAT1 and IFIT3 were highly expressed in tumors with high CD8 infiltration levels but lowly expressed in tumors with low CD8 infiltration levels. These results suggest that two genes may influence the role of CD8^+^ T cells in GC immunotherapy.

### Association of hub genes variants with prognosis

Many studies have shown that individual altered genes affect the survival and response to ICIs in multiple types of tumors (12, 47). The distributions of SNVs from the TCGA-STAD cohort showed that altered frequencies of STAT1 and IFIT3 came first and third among 48 gastric cancer patients (**Figure 4SF**). Similarly, the altered frequencies of STAT1 and IFIT3 came second and fourth among 525 samples from the pan-cancer TCGA cohort (**Figure 4SF**). TIMER web serve allowed us to assess the relationship between six immune cell infiltrates and hub gene somatic copy number variations (CNVs) according to the TCGA-STAD dataset (48, 49). It was found that there were significant interactions between five various CNVs of each hub gene and the infiltration level of six immune cells (all p<0.05; **Figure S5A**). Moreover, heterozygous amplification was found to dominate in uterine carcinosarcoma (UCS), esophageal carcinoma (ESCA), adrenocortical carcinoma (ACC) and testicular germ cell tumors (TGCT); and heterozygous deletion was present in ovarian serous cystadenocarcinoma (OV) and breast invasive carcinoma (BRCA, **Figure S5B**). Heterozygous amplification and heterozygous deletion were the top two presents in the TCGA-STAD dataset.

Next, survival analysis of pan-cancer showed that hub genes might be risk factors of survival for five TCGA cohorts, including LGG, kidney renal papillary cell carcinoma (KIRP), kidney renal clear cell carcinoma (KIRC), uveal melanoma (UVM) and thymoma (THYM; HR > 1, p< 0.05; **Figure S6A**), while the opposite tendency was found for the SKCM cohort (HR<1, P<0.05). Hub genes mainly inhibited apoptosis, EMT, and ER hormone signaling pathways and activated RTK, AR Hormone, PI3KAKT and TSCmTOR signaling pathways in pan-cancer cohort (**Figure S6B**). Moreover, Hub genes expression had significantly positive correlations with the frequencies of CNV in most diverse cancer types, implying that CNV may positively regulate the hub genes expression in the process of invasion, metastasis and recurrence of a variety of tumors (**Figure S6C**).

### A predictive model for prognosis and immunotherapy response

**Figure 5:**
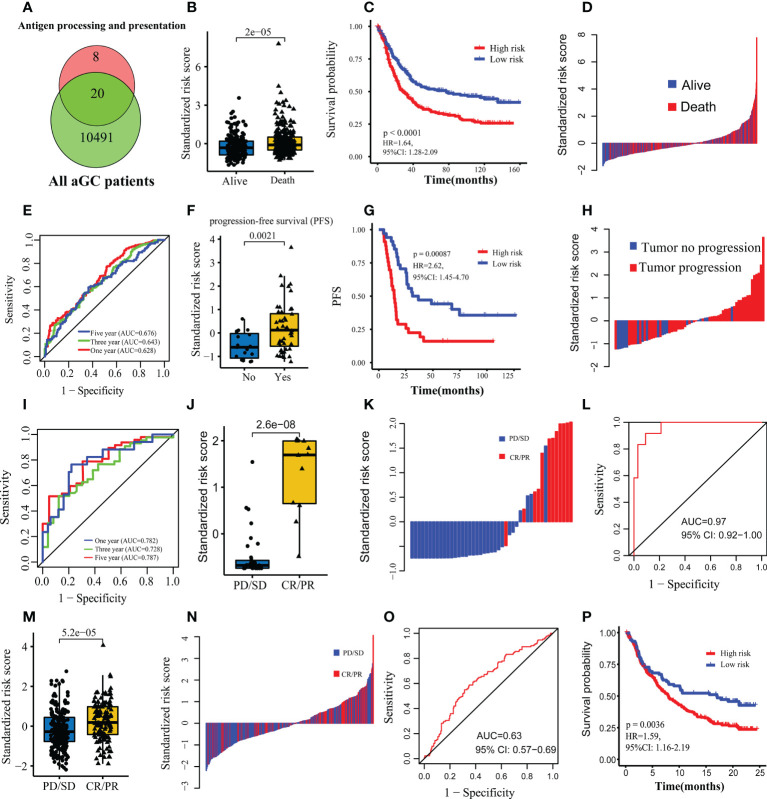
Construction and validation of the predictive model for prognosis and immunotherapy response. **(A)** Venn map showing hub genes used to construct the predictive model. **(B)** Comparison of the standardized risk score between dead and alive patients in FMAC. **(C)** The Kaplan-Meier survival curves between the high-risk and low-risk patients in FMAC. **(D)** Standardized risk score distribution plot in FMAC. **(E)** ROC for the risk score predicting prognosis in FMAC. **(F)** Comparison of the standardized risk score between tumor progression and tumor progression-free patients in the GSE26253 dataset. **(G)** The Kaplan-Meier progression-free survival (PFS) curves between high-risk and low-risk patients in GSE26253 dataset. **(H)** Standardized risk score distribution plot in GSE26253 dataset. **(I)** ROC for the risk score predicting prognosis in GSE26253 dataset. **(J)** Comparison of the standardized risk score between patients with immunotherapy response and patients without immunotherapy response in the Kim cohort. **(K)** Standardized risk score distribution plot in the Kim cohort. **(L)** ROC for the risk score predicting immunotherapy response in the Kim cohort. **(M)** Comparison of the standardized risk score between patients with immunotherapy response and patients without immunotherapy response in the IMvigor210 cohort. **(N)** Standardized risk score distribution plot in the IMvigor210 cohort. **(O)** ROC for the risk score predicting immunotherapy response in the IMvigor210 cohort. **(P)** The Kaplan-Meier survival curves between the high-risk and low-risk patients in the IMvigor210 cohort.

By intersecting 28 genes being enriched in antigen processing and presentation (**Figures 1F, G**), with 10511 genes from 467 patients in FMAC, 20 candidate genes were obtained (**Figure 5A**). After stepwise backward Cox regression, the final predictive model consisting of nine genes (B2M, CTSB, HLA-A, HLA-C, HLA-DRA, HLA-F, HSPA2, KLRD1 and TAPBP) was constructed. The formula of the predictive model calculated the risk score of each patient. Risk scores in dead patients were significantly higher than that in alive patients (p=2e−05, **Figure 5B**). Those patients with risk scores exceeding the median risk score were classified as high-risk patients. On the contrary, other patients were classified as low-risk patients. The survival of high-risk patients was poorer than low-risk patients (p< 0.0001, **Figure 5C**). In particular, the risk score could identify dead patients from alive patients (AUCs for one-year, three-year, and five-year survival were 0.628, 0.643 and 0.676, respectively; **Figures 5D, E**).

Next, we used the GSE26253 dataset to validate the predictive model for prognosis and immunotherapy response and found that risk scores in tumor progression samples were significantly higher than those of tumor progression-free samples (p= 0.0021, **Figure 5F**). The PFS of high-risk patients was poorer than low-risk patients (log-rank test, p=0.00087, **Figure 5G**). Additionally, the risk score identified tumor progression samples from tumor progression-free samples (AUCs for one-year, three-year, and five-year survival were 0.782, 0.728 and 0.787, respectively; **Figures 5H, I**).

To further explore whether the above nine genes can predict the response to ICIs in the Kim cohort, the risk score of each patient was calculated using the formula of logistic regression analyses. Expectedly, patients with response to ICIs had higher risk scores than those without response to ICIs (p=2.6e−08, **Figure 5J**). Of note, the risk score perfectly discriminated patients with vs. without response to ICIs as evidenced from the ROC curve and corresponding high AUC value (AUC=0.97, 95% CI: 0.92−1.00; **Figures 5K, L**). Moreover, we used the IMvigor210 cohort of BC to estimate the ability of the risk score to predict response to ICIs. Consistent with the results of the Kim cohort, risk scores in BC patients with response to ICIs were significantly higher than those without response to ICIs (p=5.2e−05, **Figure 5M**). The risk score also could discriminate BC patients with vs. without response to ICIs (AUC=0.63, 95% CI: 0.57−0.69; **Figures 5N, O**). Lastly, the survival of high-risk patients was poorer than low-risk patients (p=0.0036, **Figure 5P**) for the IMvigor210 cohort. In brief, the risk score consisted of nine genes associated with antigen processing and presentation is an effective indicator of prognosis and response to ICIs for aGC patients.

### Predictive model genes for prognosis and immunotherapy response

**Figure 6:**
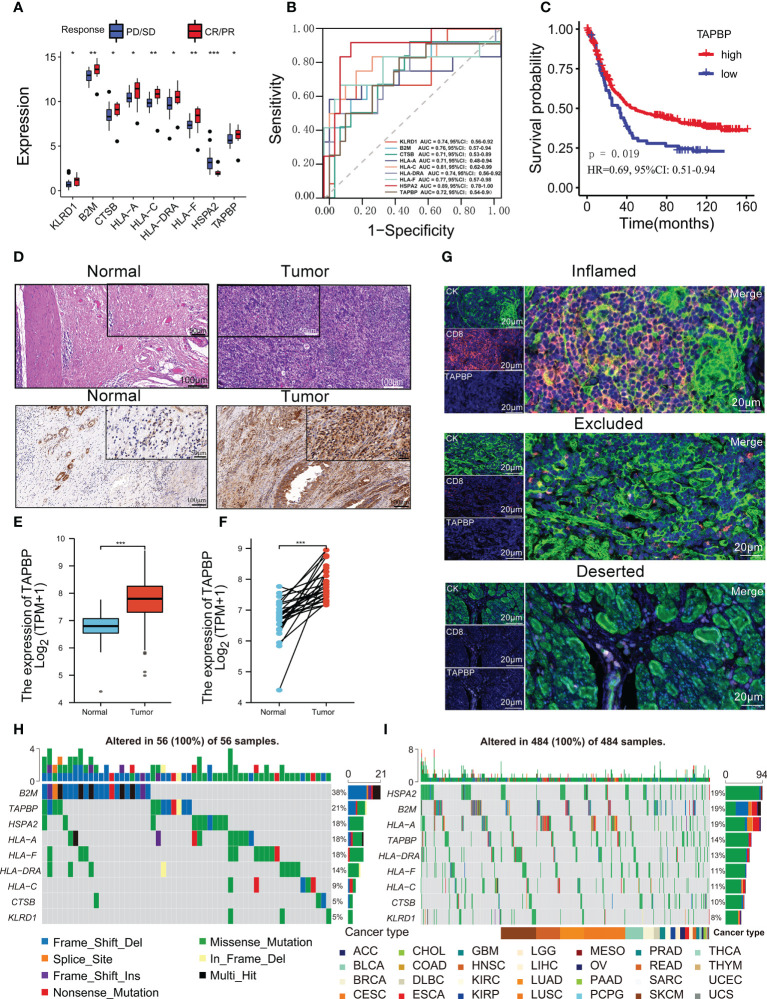
TAPBP was related to the immunotherapy response and the tumor immune microenvironment. **(A)** Comparison of nine predictive model genes between CR/PR group and SD/PD group in the Kim cohort. **(B)** ROCs for nine genes predicting immunotherapy response in the Kim cohort. **(C)** The Kaplan-Meier survival curves between the Kim cohort’s high-TAPBP expression group and the low-TAPBP expression group. **(D)** HE and IHC staining results in tumor and normal tissues in GC. The expression of TAPBP in between tumor tissues and normal tissues **(E)** or adjacent normal tissues **(F)** from the TCGA-STAD cohort. **(G)** The multi-color fluorescence staining detected the spatial distribution of TAPBP and CD8 in the immune-inflamed subtype, immune-excluded subtype, and immune-deserted subtype of GC. The distributions of nine predictive model genes somatic alterations from TCGA-STAD cohort **(H)** and pan-cancer TCGA cohort **(I)**. Asterisks denote significant differences (*p< 0.05; **p< 0.01; ***p< 0.001).

In the Kim cohort, patients with response to ICIs had lower HSPA2 expression than that without response to ICIs, whereas the opposite was observed for the other eight genes (all p<0.05, **Figure 6A**). Of nine genes, eight could discriminate patients with vs. without response to ICIs except for HLA-A (**Figure 6B**).

We further assessed the correlations between nine predictive model genes and 22 immune cells (**Figure S7A**). Eight genes (TAPBP, KLRD1, HSPA2, HLA-DRA, CTSB, B2M, HLA-C and HLA-A) were significantly associated with more than nine types of immune cells in 467 patients (all p<0.05). This result implied that nine genes might regulate immune cell infiltration in tumor tissue. Notably, TAPBP was significantly correlated with most immune cells (n=16) and was positively correlated with CD8 T cells, activated memory CD4 T cells and activated NK cells, which could kill tumor cells. The MCP-counter was used to estimate the absolute abundance of eight immune and two stromal cell populations in GC tissues from TCGA-STAD transcriptomic data, showing that TAPBP had positive relationships with most immune cells except for neutrophils (**Figure S7B**). Additionally, the survival of patients with high TAPBP expression was better than low TAPBP expression, implying that this gene might be a protective factor (p = 0.019, **Figure 6C**). The hematoxylin-eosin staining (HE) showed that the infiltrating gastric carcinoma cells in tumor tissues were higher than in adjacent tissues (**Figure 6D**). Moreover, immunohistochemistry (IHC) was used to measure the expression of TAPBP protein in tumor tissues and adjacent normal tissues. The staining intensity score of TAPBP in tumor tissues was significantly higher than that in adjacent normal tissues (**Figure 6D**). According to the TCGA-STAD cohort, it was found that the expression of TAPBP mRNA in tumor tissues was higher than that in normal tissues (Mann-Whitney U test, p<0.0001; **Figure 6E**). Pair Wilcoxon signed-rank test also suggested that the expression of TAPBP mRNA in tumor tissues was higher than in adjacent normal tissues (p<0.0001; **Figure 6F**). The multi-color fluorescence staining revealed the highest expression of TAPBP and CD8 in the immune-inflamed subtype, followed by the immune-excluded subtype and immune-deserted subtype (**Figure 6G**).

**Figure 7:**
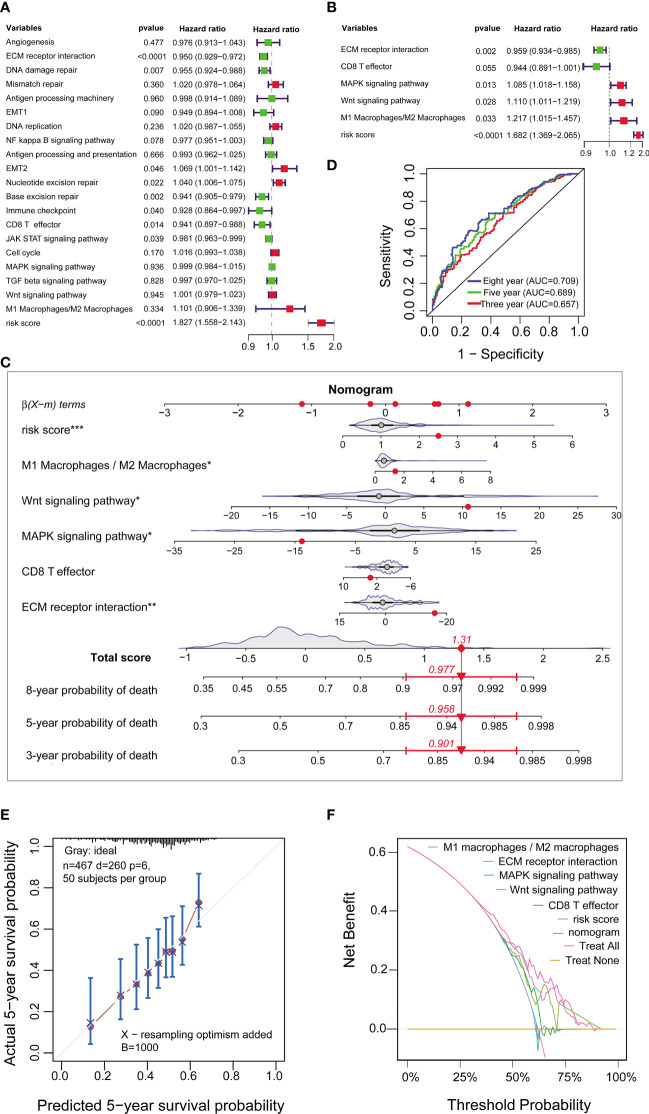
Construction of a nomogram for prognosis in FMAC. **(A)** The univariate and **(B)** multivariable Cox regression for the risk score and essential signaling pathways in FMAC. **(C)** The nomogram constructed by six variables. The red dot represents the risk score corresponding to the value of the independent variable. According to the total scores of the nomogram, the three-year, five-year and eight-year death probability in randomly selected the 400th patient being 0.901, 0.958 and 0.977, respectively. **(D)** ROCs for three-year, five-year and eight-year death probability of the nomogram. **(E)** Calibration curves of the predicted five-year survival probability and actual five-year survival probability. The gray line indicates ideal fit; the circle indicates the predicted five-year survival probability of nomogram; the stars indicate bootstrap correction estimates, and the error bars indicate the 95% CI of these estimates. **(F)** Decision curves for the nomogram, the risk score of nine prediction model genes and other four factors.

Moreover, patients with high HLA-C, HLA-DRA, HLA-F and KLRD1 expression had better survival, while the opposite trend was observed for high B2M, CTSB, HLA-A and HSPA2 expression (**Figure 7SC**). We used 1146 individuals with more stage III from 16 independent TCGA cohorts to assess the prognostic value of TAPBP. Although TAPBP was not significantly associated with survival in 16 types of tumors, the meta-analysis showed that patients with high-expression TAPBP had better survival than low-expression TAPBP (p<0.0001, **Figure 8SA**).

The distributions of somatic alterations from the TCGA-STAD cohort showed that altered frequencies of B2M and TAPBP came top two among 56 GC patients (**Figure 6H**). Similarly, the altered frequencies of B2M and TAPBP came first and fourth among 484 samples from pan-cancer TCGA cohort (**Figure 6I**). We further analyzed the difference of nine predictive model genes between subtypes in the pan-cancer TCGA cohort, and found six types of hot cancers including BRCA, lung adenocarcinoma (LUAD), glioblastoma multiforme (GBM), KIRC, lung squamous cell carcinoma (LUSC) and HNSC were the most significant (**Figure 8SB**). KIRC has the largest fold difference of nine predictive model genes expression between tumor and normal tissue (**Figure S8C**). Similar changes in nine predictive model genes were observed for BRCA and HNSC, whereas the opposite was observed for LUSC. The heatmap showed that nine predictive model genes initiated apoptosis, EMT, hormones ER and RAS/MAPK signaling pathways, while the opposite function was observed in cell cycle, hormones AR and DNA damage response signaling pathways (**Figure S8D**). The above results revealed that nine predictive model genes might interact with immune cells in the tumor microenvironment to affect a variety of cancers invasion, metastasis and recurrence.

### A nomogram for prognosis

The univariate Cox analysis identified the risk score of nine prediction model genes and nine signaling pathways, including ECM receptor interaction, DNA damage repair, EMT2, Nucleotide excision repair, Base excision repair, Immune checkpoint, CD8 T effector and JAK-STAT signaling pathways, significantly associated with the prognosis of GC patients (all p<0.05; **Figure 7A**). The multivariate Cox analysis further showed that the risk score of nine prediction model genes, MAPK signaling pathway, Wnt signaling pathway, the ratio of M1 Macrophages to M2 macrophages were risk factors of the survival in GC, while ECM receptor was a predictive factor (all p<0.05; **Figure 7B**). Although CD8 T effector did not have a significant association with the survival (p =0.055), it was an essential indicator of immunotherapy and was enter into the multivariate Cox analysis.

Next, we built a nomogram model of six significant variables in the multivariate Cox regression (**Figure 7C**). The AUCs for predicting three-year, five-year, and eight-year survival of GC patients were 0.657, 0.689 and 0.709, respectively (**Figure 7D**). When compared to previously published prognostic biomarkers of various measurements, including mRNA expression, lncRNA expression, microRNA expression, protein, and DNA methylation level for predicting the prognosis of GC patients (50–55), the AUC of our nomogram model for predicting eight-year survival appeared to be higher. The capacity to predict outcomes for more patients is likely increased by the addition of more clinical characteristics to our model, or it may be facilitated by the inclusion of more samples and a longer period of follow-up in our study. Calibration curves showed that the predicted 5-year survival probability and actual 5-year survival probability were hovering near the 45-degree diagonal line, implying that the nomogram model has a superior ability to predict GC prognosis (**Figure 7E**). Moreover, decision curve analysis showed that the curves of the nomogram model and risk score of nine prediction model genes were isolated from angles of treatment for all and treatment for none, highlighting the clinical potential of the nomogram model in the prognosis of aGC (**Figure 7F**).

## Discussion

The antigen processing and presentation plays a crucial role in effector T cells recognition of tumor cells, influencing the identification of patients susceptible to immunotherapy (11). This study identified a novel biomarker of genes related to antigen processing and presentation, predicting response to ICIs for aGC patients. Currently, most studies use TCGA or GEO datasets to build models for predicting prognosis rather than response to ICIs for aGC patients due to the lack of ICIs treatment results. Therefore, we explored the response to ICIs for aGC patients in the Kim cohort treated by ICIs to make the results have direct clinical translational significance.

In this study, we built a scoring system to evaluate the efficacy of immunotherapy for aGC patients. We validated the results—the APscore distinguished patients with or without response to ICIs in the Kim cohort. Moreover, patients with response to ICIs had higher APscore than those without response to ICIs. Several subtypes of GC, such as high MSI subtype, positive EBV subtype, high TMB subtype and high immune signature subtype, benefited from ICIs treatment (5). These subtypes also had high Apscore in the Kim cohort, further revealing that APscore might effectively predict immunotherapy efficacy. Thus, the critical biomarkers involved in the mechanism of immunotherapy need to be identified and assessed.

Ten hub genes, STAT1, IRF1, ISG15, IRF9, BST2, PSMB8, STAT2, IFI35, IFIT3 and OAS2 from 366 antigen processing and presentation-related genes were further analyzed. The Metascape shows that ten hub genes are enriched in interferon-alpha/beta signaling, which has been reported to influence APM gene expression (56, 57). This result suggests that we can enhance antigen presentation efficiency of initially unresponsive patients with ICIs by stimulating interferon signal transduction to improve immunotherapy efficacy, especially in aGC patients with low APscore, which has potential transformation significance. A large body of evidence has been provided about STAT1 inhibiting proliferation and promoting apoptosis of tumor cells, and it is considered a potential tumor inhibitor (58, 59). STAT can induce tumor cell apoptosis by interfering with the MARK signaling pathway or down-regulating interleukin (IL-6) expression and STAT3 of interferon-alpha/beta signaling (60, 61). Furthermore, new studies show that down-regulated STAT1 expression in peripheral blood of GC patients results in immune escape from gastric epithelial cancer caused by decreased anti-tumor immune function (62, 63). However, there are few experimental studies on the interaction between immune microenvironment and tumor antigenicity and antigen presentation in GC immunotherapy. This study used multi-color immunofluorescence staining to accurately locate positive spatial associations of IFIT3 and STAT1 expression with PD-L1 and CD8 expression, representative immunotherapy targets in different immunotypes of aGC tissues. Hub genes selected to share a common pathway enrichment of interferon signaling pathway, and the relationship between specific genes and immunotherapy may provide new insight into the ICB therapy for GC.

According to nine genes (B2M, CTSB, HLA-A, HLA-C, HLA-DRA, HLA-F, HSPA2, KLRD1 and TAPBP) enriched in antigen processing and presentation, we built a predictive survival model for aGC patients with high accuracy and sensitivity. In addition, a nomogram based on the risk score and several signatures of critical signaling pathways was plotted and showed that the risk score had robust survival prediction ability. Currently, the relationships between nine genes and the survival of various tumors may be easily explored using TCGA or GEO data. However, how they affect the immunotherapy efficiency of GC has rarely been studied. We found that the prediction model of the above nine genes predicted response to ICIs in aGC patients with a value of AUC 0.97. These results suggest that the nine genes are valuable for further study in the immunotherapy mechanism of GC. In addition, several other genes have already been linked to cancer-specific immune responses. For example, mutation of B2M gene resulted in defective expression of MHC-1 molecular antigen, which could not deliver antigen to CD8^+^T cells through TCR and disturbed the positive selection of CD8 cells in the thymus to affect the development of CD8 T cells (64, 65). Several studies showed that HLA-I, including HLA-A, HLA-B and HLA-C positive tumors may be more susceptible to immune checkpoint inhibitors (66, 67).

In contrast, HLA-I negative tumors might be associated with acquired drug resistance to PD-1 blockade in cancer patients. It was found that the increased cell apoptosis in gastric cancer leads to the release and degradation of CTSB protein from the lysosome, resulting in gastric cancer cell death (68). The cleavage of Caspase-1 by CTSB activates caspase-1, which promotes the secretion of interleukin-1β (IL-1β), leading to an inflammatory response (69, 70). Compared with atezolizumab monotherapy for biliary tract cancer, TAPBP expression was higher in the combined treatment group (71). Compared with attezolzumab monotherapy for biliary tract cancer, TAPBP expression was higher in the combined treatment group (atezolizumab + MEK inhibitor). As an important biomarker, TAPBP from TIGS is an effective intrinsic tumor biomarker and can predict ICIs response in pan-cancers (10). The TAPBP effectively predicted immunotherapy response in aGC patients and stratified patients into groups with significantly different survival in this study. In addition, the positive correlation between TAPBP expression and tumor microenvironment was confirmed by multi-color immunofluorescence staining, providing a clue for further study on the effect of TAPBP on ICB therapy.

There are some limitations to this study. First, we did not conduct external validation because it was challenging to gather clinical patient data for ICIs treatment in aGC patients. Nonetheless, 467 patients from TCGA and GEO datasets were used to validate our results by estimating immunotherapy response by TIDE. Second, even though IHC and fluorescence experiments were carried out to validate the model results, large-scale protein sequencing analysis would be a better choice. Third, we analyzed the effects of APscore on the immune microenvironment, SNV, TMB and signaling pathways, but the underlying mechanism remained unclear. Therefore, to better understand the significance of APscore in predicting GC immunotherapy response, further *in vivo* and *in vitro* experimental research are required.

In summary, our work presents for the first time a novel signature based on genes associated with antigen processing and presentation, that can help identify response to immunotherapy, allow identification of high-risk patients and predict prognosis in aGC patients. Further studies using gene expression profiles may represent a powerful approach to explore the biological mechanism of tumor immunotherapy escape, and may provide new targets for transforming anti-PD-1 resistant tumors into a responsive state.

## Data availability statement

The raw data supporting the conclusions of this article will be made available by the authors, without undue reservation.

## Ethics statement

This study was reviewed and approved by the ethics committee of affiliated hospital of Jiangnan University (approval number: LS2020050). Written informed consent was obtained from all participants for their participation in this study.

## Authors contributions

KW and FY conceived the study. MW, BH, XW and QY participated in study selection, data extraction and statistical analysis. MW and ZL performed Hematoxylin-eosin staining (HE) and organized sample data. BH provided FFPE tumor samples. KW, ZY and JW made the figures. KW and FY wrote the first draft of the manuscript. All authors interpreted the results, and approved the final version for submission.

## Funding

This work was supported by the top Talent Support Program for young and middle-aged people of Wuxi Health Committee (No. HB2020040), Mega-project of Wuxi Commission of Health (No. Z202007).

## Acknowledgments

We thank Gene-Expression Omnibus (GEO) and the Cancer Genome Atlas (TCGA) database for providing the transcriptome and clinical information. We want to thank the pathology department of the affiliated hospital of Jiangnan University for their technical support and Yankui Liu’s help, the technical engineer.

## Conflict of interest

The authors declare that the research was conducted in the absence of any commercial or financial relationships that could be construed as a potential conflict of interest.

## Publisher’s note

All claims expressed in this article are solely those of the authors and do not necessarily represent those of their affiliated organizations, or those of the publisher, the editors and the reviewers. Any product that may be evaluated in this article, or claim that may be made by its manufacturer, is not guaranteed or endorsed by the publisher.

## Glossary

**Table:**

GC	gastric cancer
ICI	immune checkpoint inhibitor
CTLA-4	cytotoxic T-lymphocyte-associated protein 4
PD-1	programmed cell death 1
PD-L1	programmed cell death-ligand 1
TMB	tumor mutation burden
TCGA	The Cancer Genome Atlas
GEO	Gene Expression Omnibus;
FMAC	final merged aGC cohort
MSI	microsatellite instability
EBV	Epstein‐Barr virus
SNV	single nucleotide variant
BC	bladder cancer
PR	Partial response
CR	complete response
PD	progressive disease
SD	stable disease
WGCNA	Weighted Gene Co-expression Network Analysis
GO	Gene Ontology
KEGG	Kyoto Encyclopedia of Genes and Genomes
PCA	principal component analysis
EMT	epithelial-mesenchymal transition;
DEGs	Differentially expressed genes
TIDE	Tumor immune dysfunction and exclusion
ROC	receiver operating characteristic curve
FFPE	Formalin fixation and paraffin embedding
HE	Hematoxylin-eosin staining
IHC	Immuno-histochemistry
MHC	major histocompatibility complex
ICB	Immune checkpoint blockade
AUC	area under curve
HR	Hazard ratio;
CI	confidence interval
Tregs	regulatory T cells
BRCA	breast invasive carcinoma
HNSC	head and neck squamous cell carcinoma
LGG	brain lower grade glioma
SKCM	skin cutaneous melanoma
PFS	progression-free survival
CNV	copy number variation
UCS	uterine carcinosarcoma
ESCA	esophageal carcinoma
ACC	adrenocortical carcinoma
TGCT	testicular germ cell tumors
OV	ovarian serous cystadenocarcinoma
BRCA	breast invasive carcinoma
KIRP	kidney renal papillary cell carcinoma
KIRC	kidney renal clear cell carcinoma
UVM	uveal melanoma
THYM	thymoma
LUAD	lung adenocarcinoma
GBM	glioblastoma multiforme;
LUSC	lung squamous cell carcinoma
GSVA	Gene Set Variation Analysis;
AJCC	American Joint Committee on Cancer
Tfh cells	T follicular helper cells
GSEA	Gene set enrichment analysis
MSS/TP53+	intact TP53 activity;
MSS/TP53−	TP53 functional loss
TME	Tumor microenvironment.
