# Supplementary material for: An antigen processing and presentation signature for prognostic evaluation and immunotherapy selection in advanced gastric cancer
Source: Front Immunol. 2022 Oct 14;13:992060. doi: 10.3389/fimmu.2022.992060 (PMC9615473; doi:10.3389/fimmu.2022.992060)
Supplement: Supplementary file 1 [file DataSheet_1.zip › Supplementary material - 1/Figure legends.docx]

**Figure legends**

**Figure S1 WGCNA analysis and gene ontology (GO) analysis of DEGs associated with immunotherapy response.** Determination of the soft-thresholding power. The relationship of the soft-threshold with scale-free R^2^ (A) and mean connectivity (B). In the left graph, the optimal soft-thresholding power is 26 based on the scale-free network. The relationship between the gene signature and blue module genes (C) or turquoise module genes (D). GO analysis for brown module genes (E) and grey module genes (F).

**Figure S2 Identification of genes associated with antigen processing and presentation.** (A) WGCNA of 1822 DEGs which may be associated with immunotherapy response. (B) Heat maps showing the gene modules related to antigen processing and presentation. (C) The relationship between turquoise module genes and the gene signature.

**Figure S3 SNVs of different APscore groups.** (A) The relationship of the APscore with other indicators of immunotherapy response predicted by TIDE algorithm in FMAC. (B) Comparison of the APscores between true CTL.flag and false CTL.flag in FMAC. (C) ROC for the APscore predicting immunotherapy response predicted by TIDE algorithm in FMAC. (D) The relationship of the APscore with TMB in the TCGA-STAD cohort. (E) Comparison of TMB between the low-APscore and high−APscore groups in the TCGA-STAD cohort. (F) The Kaplan-Meier survival curves between the low-TMB and high-TMB groups from the TCGA-STAD cohort. (G) The Kaplan-Meier survival curves among interactions between the APscore and TMB groups. (H) The multivariate Cox regression forest plot of clinicopathological variables and the APscore. (I) Forest plot of the APscore affecting the prognosis in pan-cancer cohort. (J) Waterfall plots showing the frequencies of mutated genes in the high-APscore and low-APscore groups in the TCGA-STAD cohort.

**Figure S4 Selection and characteristics of hub genes associated with prognosis.** (A) Kaplan-Meier plots of overall survival of hub genes for the FMAC. (B) Comparison of the hub genes expression between two groups with different immunotherapy responses in the Kim cohort. (C) The multivariate Cox regression forest plot of hub genes in the GSE26253 dataset. (D) The Kaplan-Meier progression-free survival (PFS) curves of STAT1 and IFIT3 expression affecting prognosis. (E) The correlations between ten hub genes and 22 immune cells infiltration, calculated by the CIBERSORT algorithm in FMAC. (F) The distributions of hub genes somatic alterations from TCG-STAD and pan-cancer TCGA cohorts.

**Figure S5** **Association of hub genes variants with immune infiltrate in the TCGA cohort. (A)** The relationships between six immune cells infiltration and somatic copy number variations (CNVs) for hub genes. (B) Proportions of CNVs of each hub gene across 33 diverse cancer types

**Figure S6 The relationships of hub genes with survival, signaling pathway, CNVs in the pan-cancer cohorts.** (A) Univariate Cox regression consisted of Hub genes affecting the prognosis of 32 diverse cancer types. (B) The percentage of cancers in which Hub genes' mRNA expression potentially affects pathway activity. Red represents the percentage of cancers in which a given gene can activate the pathway, and inhibition is shown in blue. (C) The correlations between hub genes and CNV in 33 diverse cancer types.

**Figure S7 The correlations of nine predictive model genes with immune cells infiltration and prognosis.** (A) The CIBERSORT algorithm calculated the correlations between nine predictive model genes and 22 immune cells infiltration. (B) Based on TCGA-STAD transcriptomic data, the correlations of nine predictive model genes with eight immune and two stromal cells were calculated by the MCP-counter method. (C) Kaplan-Meier plots of high-group and low-group from eight genes-expressions for overall survival in FMAC.

**Figure S8 Pan-cancer analysis of nine predictive model genes.** (A) Forest plot of the prognostic value of TAPBP in 16 independent TCGA cohorts. (B) The associations between subtypes and TAPBP in the pan-cancer TCGA cohorts. (C) The difference of nine predictive model genes between tumor and normal tissues in the selected cancers. (D) The percentage of cancers in which nine predictive model genes have potential pathway activity effects. Red represents the percentage of cancers in which a given gene can activate the pathway, and inhibition is shown in blue.
