# Supplementary figures and images for: An antigen processing and presentation signature for prognostic evaluation and immunotherapy selection in advanced gastric cancer
Source: Front Immunol. 2022 Oct 14;13:992060. doi: 10.3389/fimmu.2022.992060 (PMC9615473; doi:10.3389/fimmu.2022.992060)

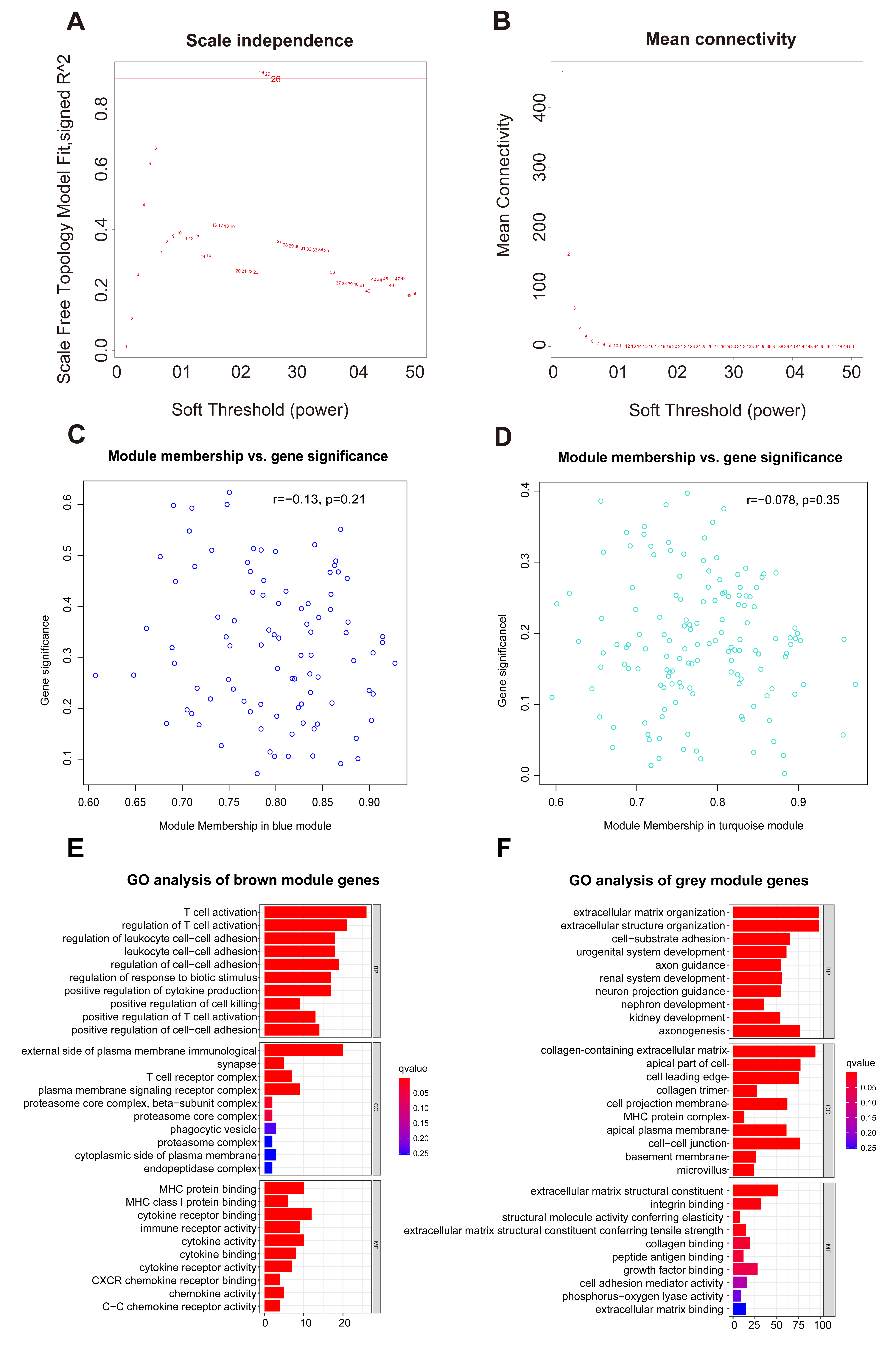

Supplement: Supplementary file 1 [file DataSheet_1.zip › Supplementary material - 1/Figure S1.tif]

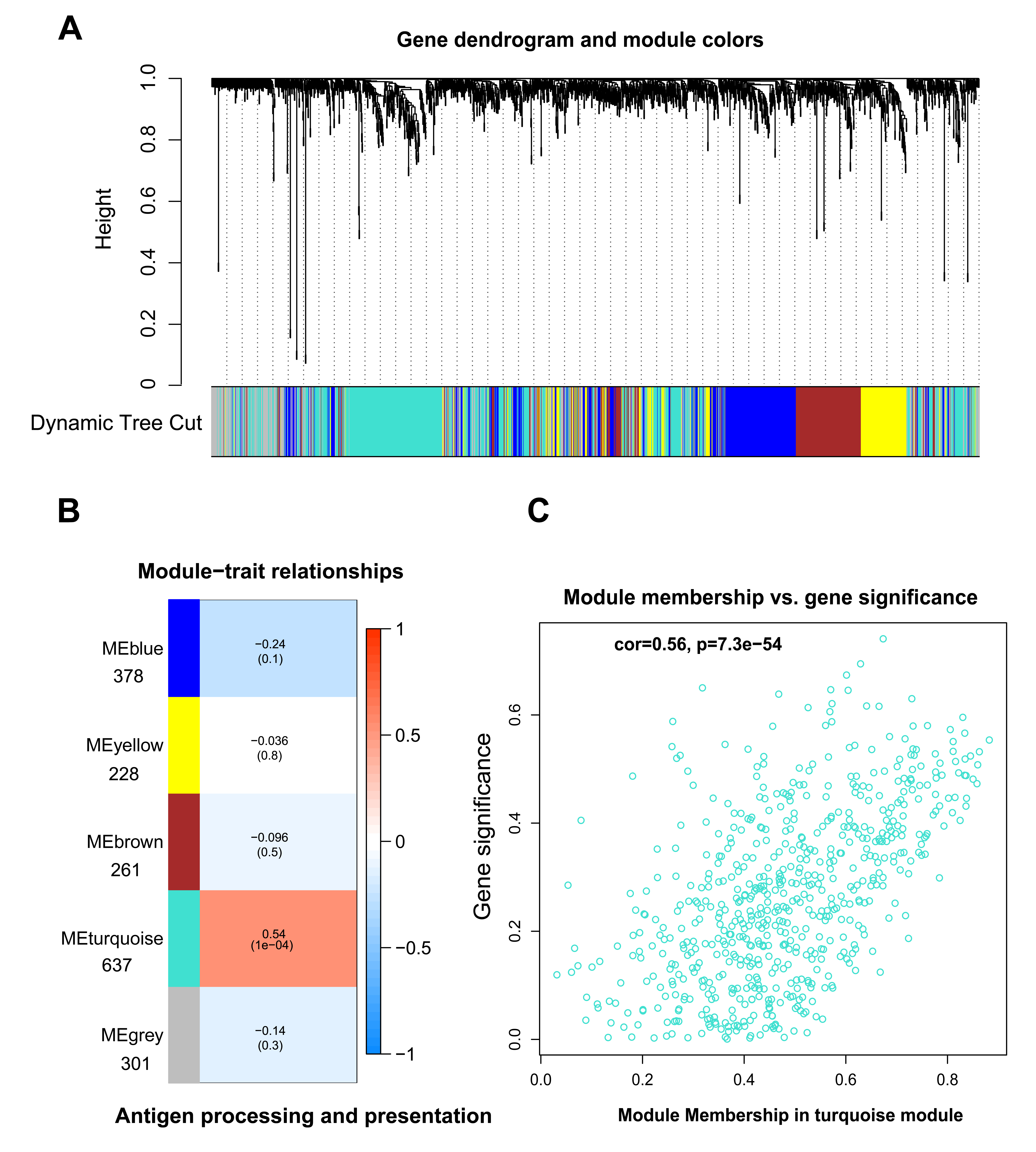

Supplement: Supplementary file 1 [file DataSheet_1.zip › Supplementary material - 1/Figure S2.tif]

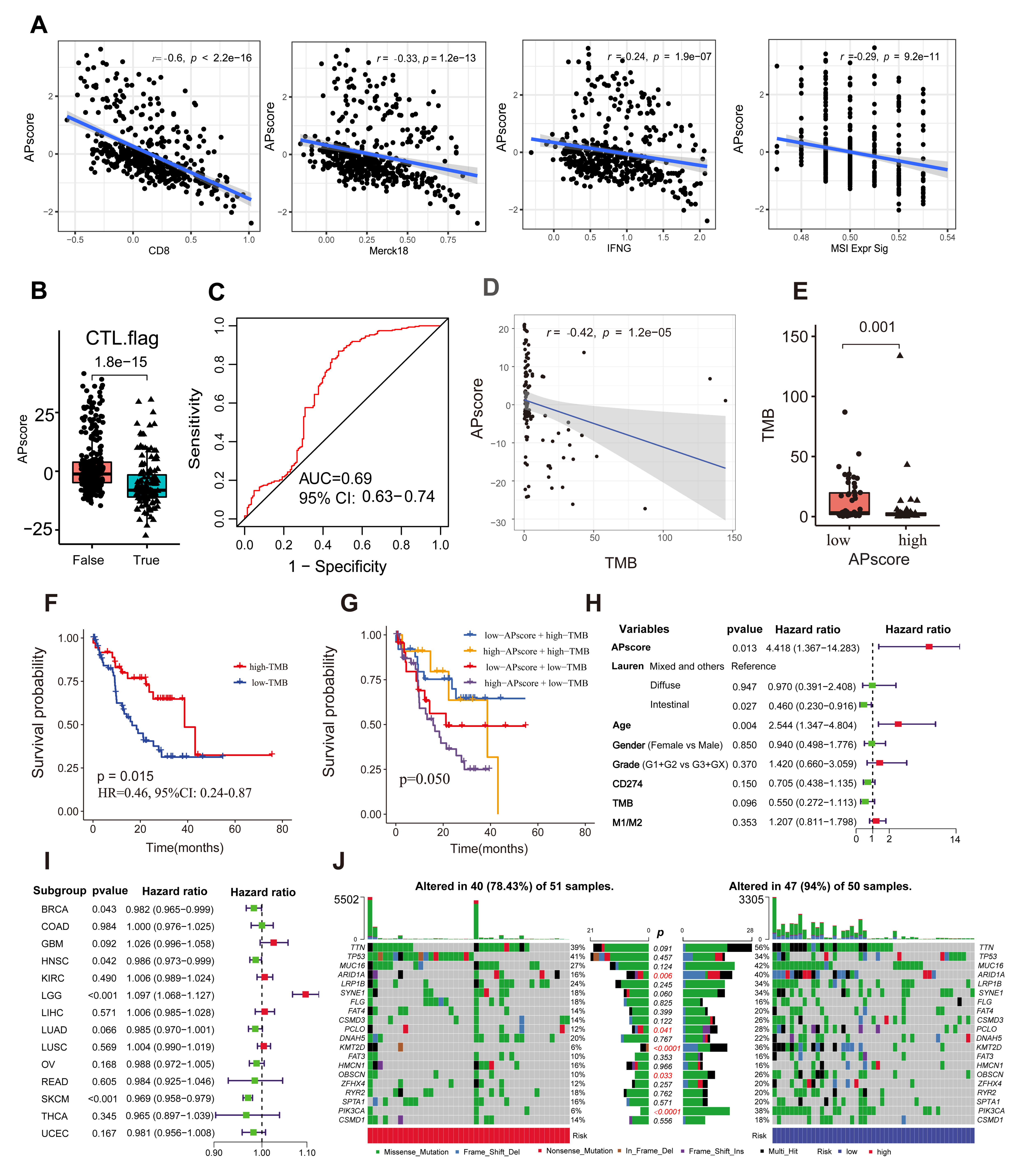

Supplement: Supplementary file 1 [file DataSheet_1.zip › Supplementary material - 1/Figure S3.tif]

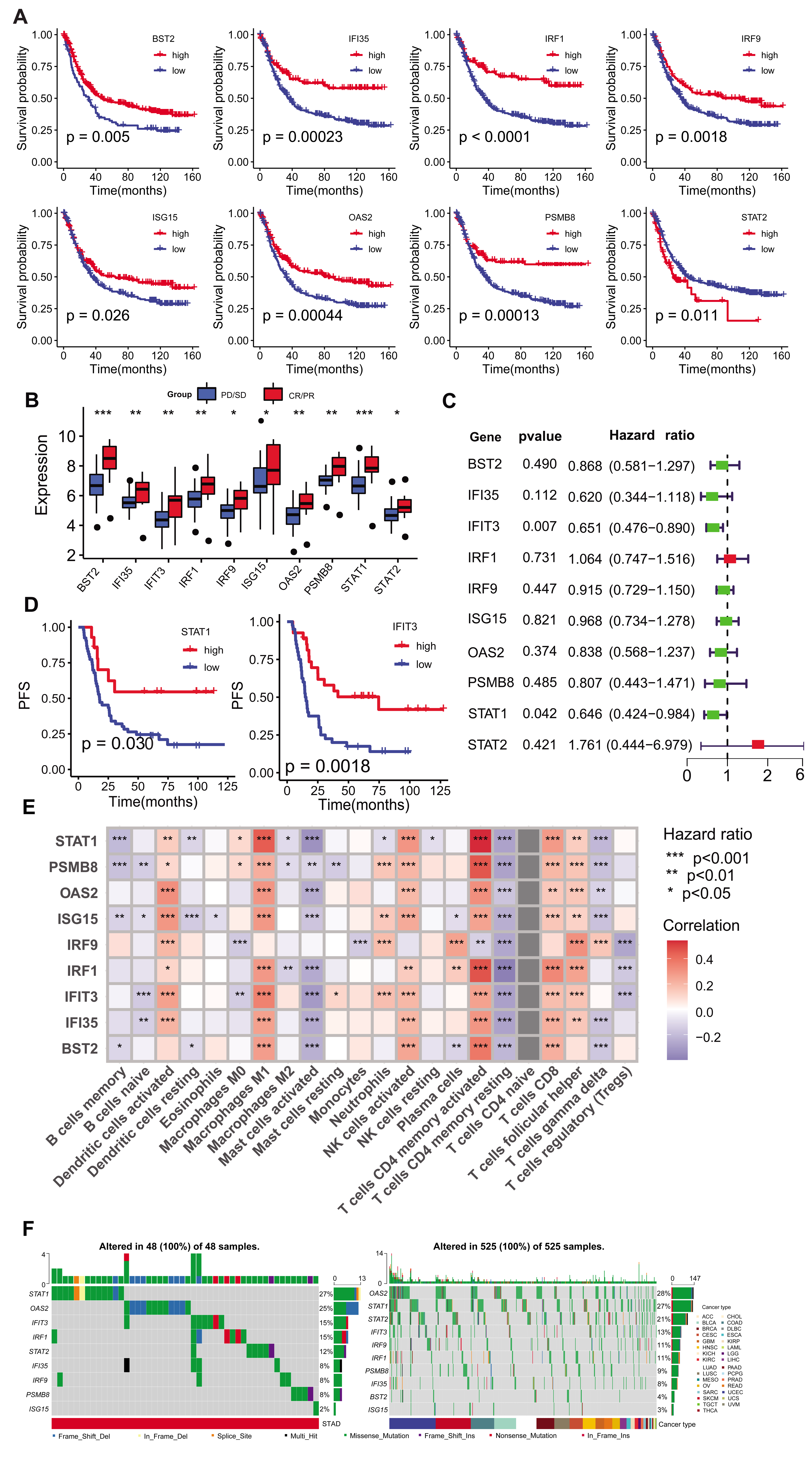

Supplement: Supplementary file 1 [file DataSheet_1.zip › Supplementary material - 1/Figure S4.tif]

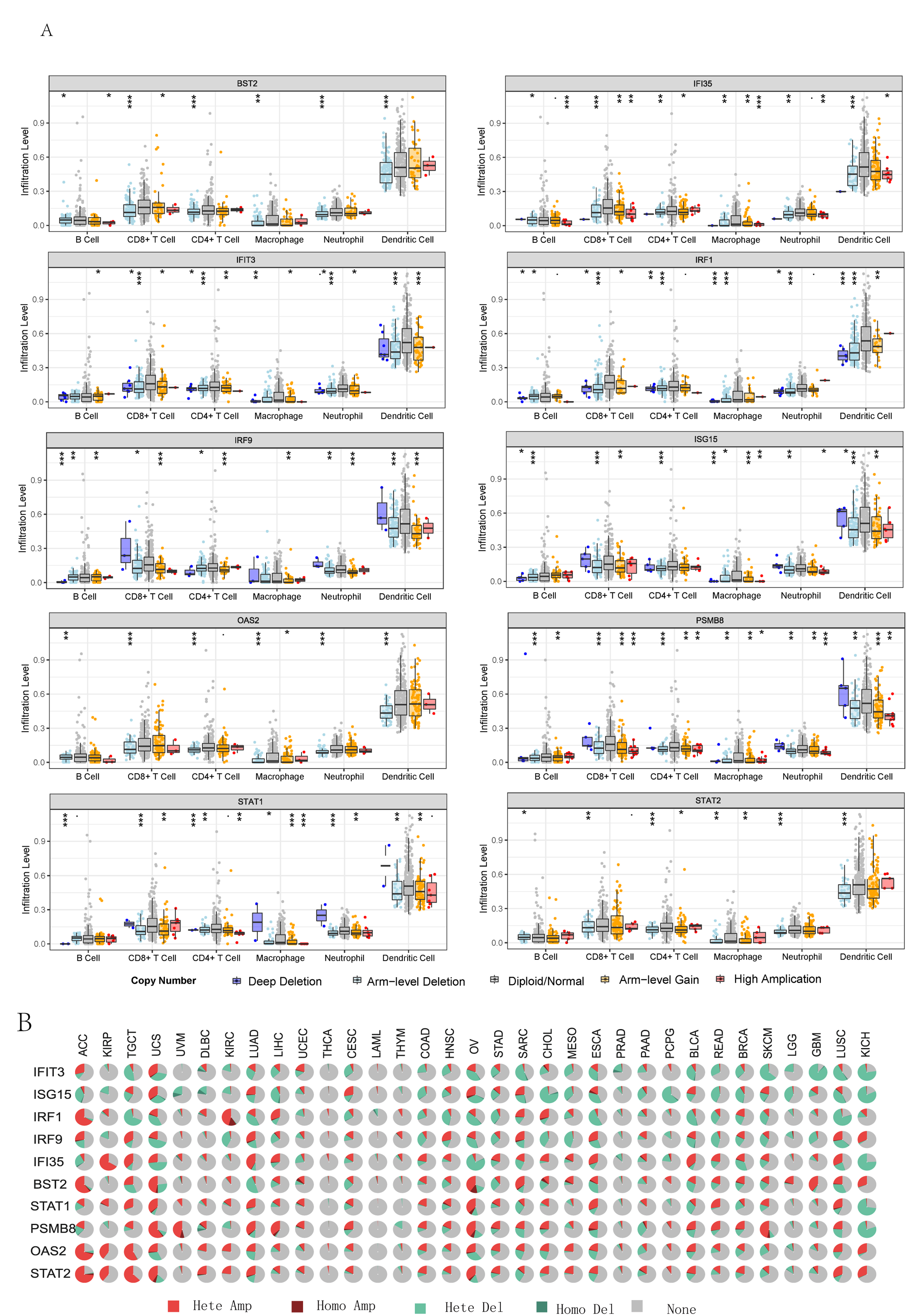

Supplement: Supplementary file 1 [file DataSheet_1.zip › Supplementary material - 1/Figure S5.jpg]

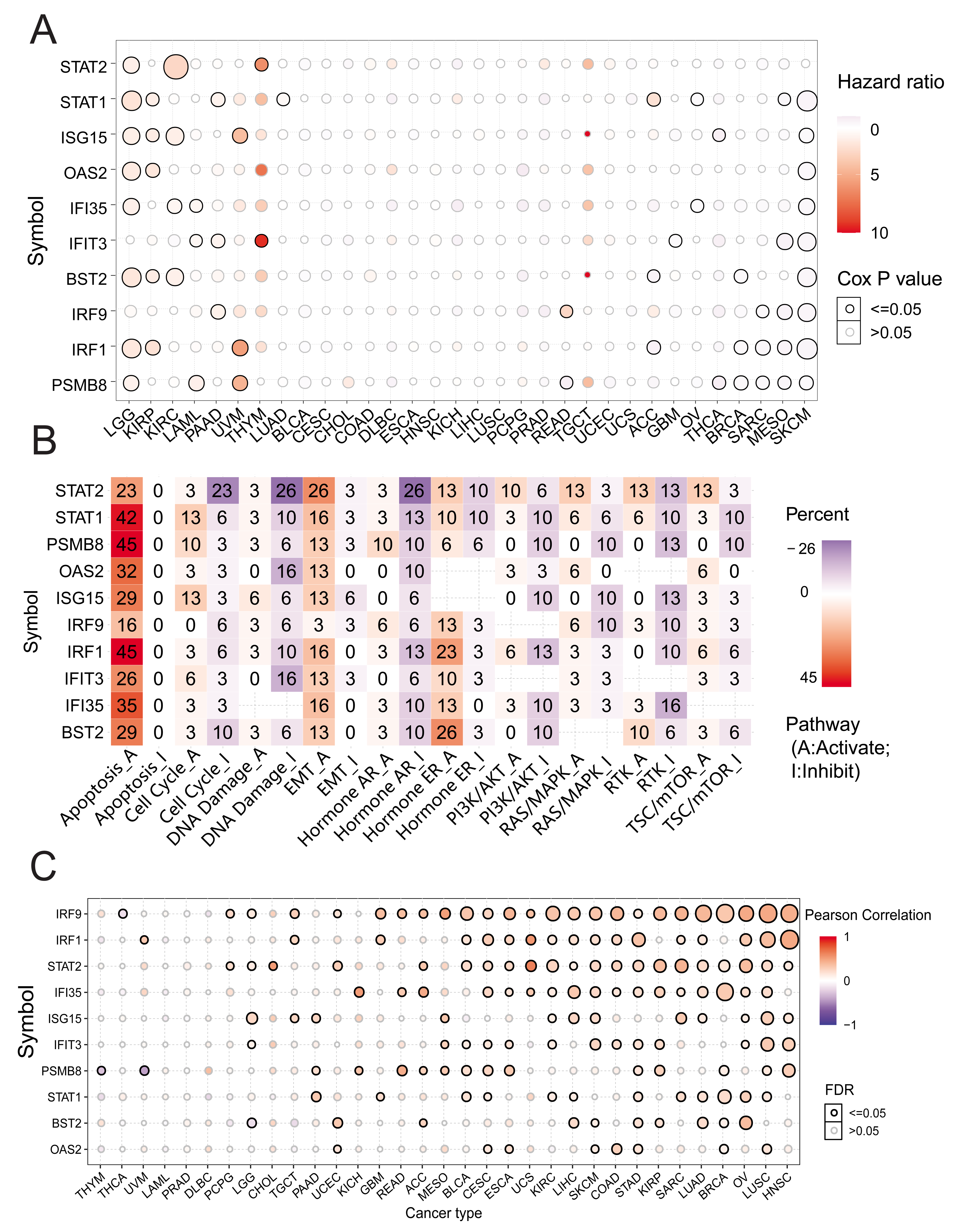

Supplement: Supplementary file 1 [file DataSheet_1.zip › Supplementary material - 1/Figure S6.tif]

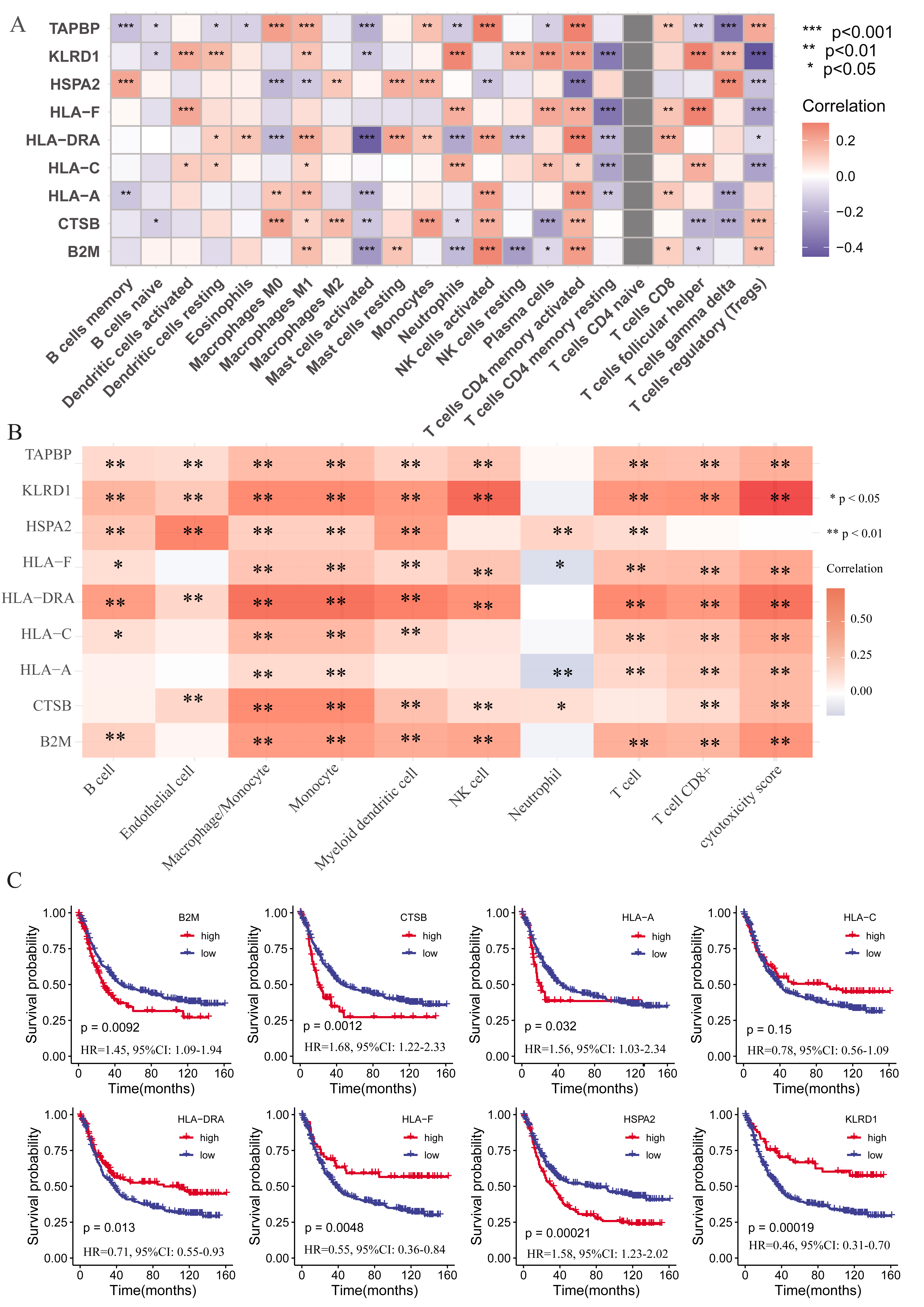

Supplement: Supplementary file 1 [file DataSheet_1.zip › Supplementary material - 1/Figure S7.tif]

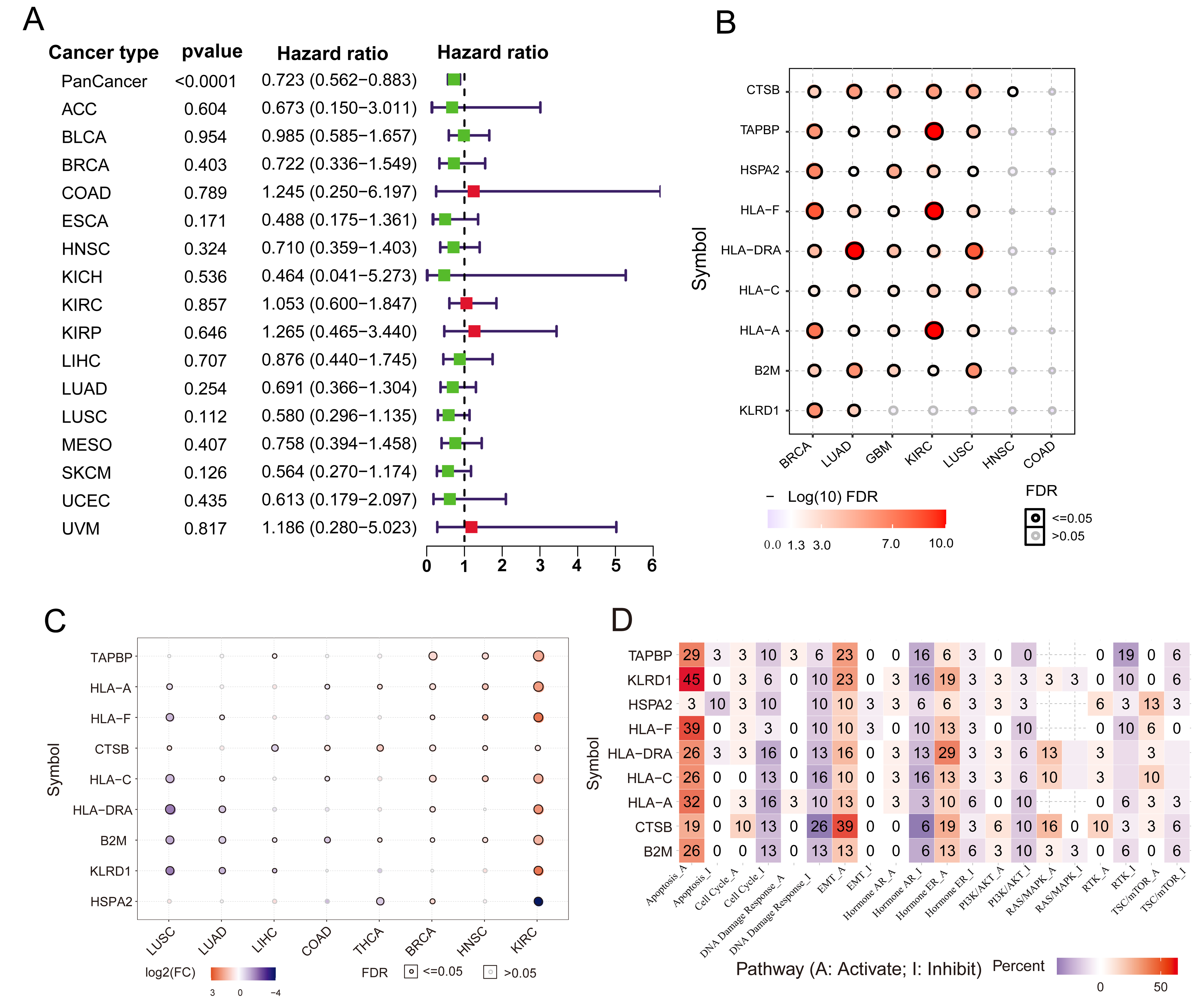

Supplement: Supplementary file 1 [file DataSheet_1.zip › Supplementary material - 1/Figure S8.tif]
